# Discovery and Characterization of Novel Bat Coronavirus Lineages from Kazakhstan

**DOI:** 10.3390/v11040356

**Published:** 2019-04-17

**Authors:** Ian H. Mendenhall, Aslan A. Kerimbayev, Vitaliy M. Strochkov, Kulyaisan T. Sultankulova, Syrym K. Kopeyev, Yvonne C.F. Su, Gavin J.D. Smith, Mukhit B. Orynbayev

**Affiliations:** 1 Programme in Emerging Infectious Diseases, Duke-NUS Medical School, Singapore 169857, Singapore; yvonne.su@duke-nus.edu.sg (Y.C.F.S.); gavin.smith@duke-nus.edu.sg (G.J.D.S.); 2 Research Institute for Biological Safety Problems, Gvardeiskiy, Kordaiskiy rayon, Zhambylskaya oblast 080409, Republic of Kazakhstan; aslan_kerim@mail.ru (A.A.K.); vstrochkov@gmail.com (V.M.S.); sultankul70@mail.ru (K.T.S.); kopeyev85@mail.ru (S.K.K.); 3 SingHealth Duke-NUS Global Health Institute, SingHealth Duke-NUS Academic Medical Centre, Singapore 168753, Singapore; 4 Duke Global Health Institute, Duke University, Durham, NC 27710, USA

**Keywords:** Kazakhstan, *Myotis blythii*, *Hypsugo savii*, coronavirus, virus discovery, phylogeny

## Abstract

Coronaviruses are positive-stranded RNA viruses that infect a variety of hosts, resulting in a range of symptoms from gastrointestinal illness to respiratory distress. Bats are reservoirs for a high diversity of coronaviruses, and focused surveillance detected several strains genetically similar to MERS-coronavirus, SARS-coronavirus, and the human coronaviruses 229E and NL63. The bat fauna of central Asia, which link China to eastern Europe, are relatively less studied than other regions of the world. Kazakhstan is the world’s ninth largest country; however, little is understood about the prevalence and diversity of bat-borne viruses. In this study, bat guano was collected from bat caves in three different sites of southern Kazakhstan that tested positive for coronaviruses. Our phylogenetic reconstruction indicates these are novel bat coronaviruses that belong to the genus *Alphacoronavirus*. In addition, two distinct lineages of Kazakhstan bat coronaviruses were detected. Both lineages are closely related to bat coronaviruses from China, France, Spain, and South Africa, suggesting that co-circulation of coronaviruses is common in multiple bat species with overlapping geographical distributions. Our study highlights the need for collaborative efforts in understudied countries to increase integrated surveillance capabilities toward better monitoring and detection of infectious diseases.

## 1. Introduction

Bats are mammals in the order Chiroptera that possess a range of unique ecological, immunological, and behavioral attributes. Bats are exceptionally speciose, comprising 20% of all mammalian species, and they are the only mammals that are capable of true flight [1]. Most bat species are gregarious and roost in large colonies, which can number over one million individuals [2]. They are relatively long-lived for their body size, and temperate species often undergo torpor or hibernation [3]. Bats also act as rich reservoirs of virus diversity with at least 23 families of viruses detected, including double-stranded DNA viruses, single-stranded DNA viruses, and positive- and negative-sense single-stranded RNA viruses [4]. Bats are incriminated as the source of several medically important virus families, including filoviruses, coronaviruses, paramyxoviruses, and reoviruses [5,6]. Several recent zoonotic spillover events and outbreaks directly or indirectly originated from bats [1].

Coronaviruses are positive-sense RNA viruses in the order Nidovirales and the family *Coronaviridae*. These viruses have the largest genome of any single-stranded RNA viruses that infect vertebrates, and they are capable of recombining to create new strains. They infect a wide variety of mammals and birds, including infectious bronchitis virus in birds and transmissible gastroenteritis virus in pigs [7]. In humans, seasonal coronaviruses can cause both upper and lower respiratory tract infections, with increased disease severity in the elderly, children, and immunocompromised patients [8]. The zoonotic SARS-coronavirus (SARS-CoV) outbreak originated in southern China from horseshoe bats, where wet markets permitted atypical contact between species, including subsequent spillover to humans [9]. Recent work showed that all genetic components of SARS-CoV co-circulate among different bat species sharing the same cave, underlying the opportunity for its re-emergence [10]. On the other hand, camels are the putative natural reservoir for MERS-coronavirus, although recent phylogenetic analysis indicated that bats harbor coronaviruses that are ancestral to the MERS-CoV lineage [11]. More recently, an HKU2-CoV outbreak caused by transmission from bats to pigs in China killed nearly 25,000 piglets [12].

Central Asia is one of the largest grassland and steppe habitats in the world, although little is known about its resident bat fauna. This habitat type is primarily located in Russia, Mongolia, and Kazakhstan. Kazakhstan is the largest land-locked country in the world; it is relatively arid (<300mm rainfall), and comprises plains and hills, with forested areas primarily restricted to the mountains in the south (Tien Shan) and the east (Alatul and Altai). Described to date, there are 27 species of bats in Kazakhstan, with 15 of these resident in Turkestan Oblast, and the most common species are *Vespertilio murinus* (Linnaeus, 1758) and *Myotis mystacinus* (Kuhl, 1817) [13,14].

While there is substantive bat research in Russia and Mongolia, there is little work on the Kazakhstan bats and less on associated virus communities [15,16]. In this study, we collected fresh bat guano from three caves at different locations in Kazakhstan and conducted molecular screening for coronaviruses. Our objective was to explore and understand the diversity of bat coronaviruses in of Kazakhstan. Here, we identified and sequenced novel bat coronaviruses and determined the evolutionary relatedness of the viruses. To the best of our knowledge, this study represents the first detection of bat coronaviruses from Kazakhstan.

## 2. Materials and Methods

**Figure 1:**
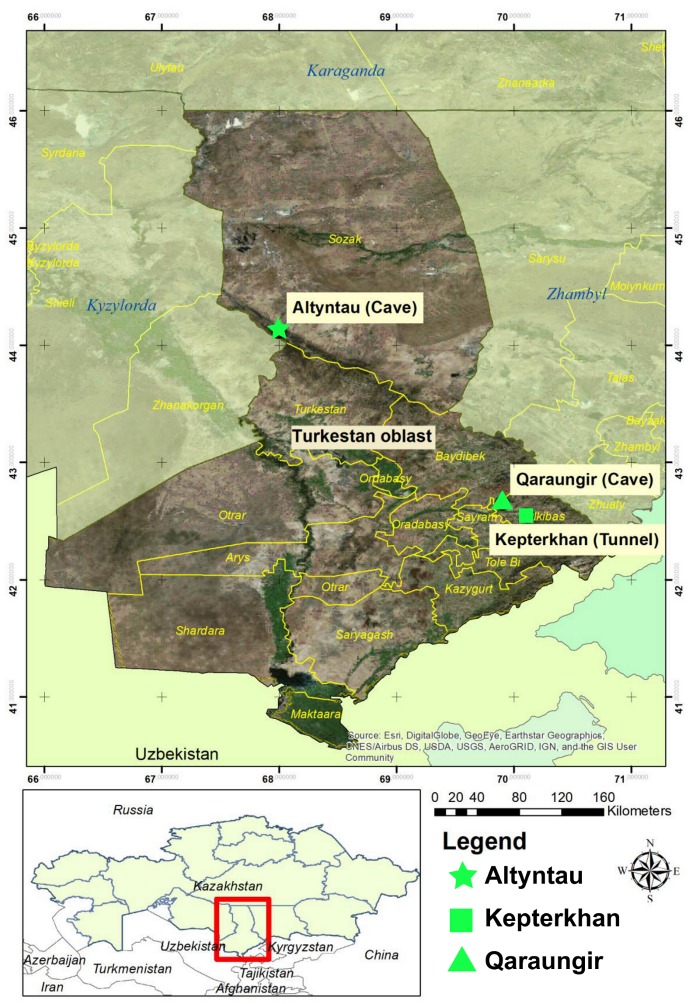
Geographical map of Kazakhstan showing the global positioning system (GPS) locations of three collection sites in this study. The green figures correspond to the sampling sites and are referenced next to the sequence name in Figure 2.

Bat guano was collected from three sites in Turkestan Oblast from 11 April to 16 May 2017. These sites were the Kepterkhan tunnel and Qaraungir cave in Tulkibas Rayon District, with additional guano collected in the Ungirli cave in Altyntau, Sozak Rayon (Figure 1). Bat feces were collected from plastic sheets placed underneath bat roosts. Multiple fecal pellets were placed into cryovials with viral transport media using polyester swabs, which were subsequently placed into a liquid-nitrogen dry shipper and then transferred back to the Research Institute for Biological Safety Problems (RIBSP) in Gvardeiskiy, Kazakhstan, where all samples were stored at −80 °C.

Bat guano was vortexed for 30 s and RNA was extracted using a QIAamp Viral RNA Mini Kit (Qiagen, Hilden, Germany), performed following the manufacturer’s instructions. Coronavirus nucleic acid was amplified using pan-coronavirus primers that amplified a 440-bp region of the RNA-dependent reverse polymerase (RdRp) (PanCor IN-6: GGTTGGGACTATCCTAAGTGTGA and PanCor IN-7: CCATCATCAGATAGAATCATCATA). A PCR was run using a Superscript III one-step RT-PCR system with Platinum Taq DNA polymerase (Invitrogen, Carlsbad, CA, USA) in a Rotorgene thermocycler (Qiagen, Hilden, Germany). The reaction consisted of 2 μL of RNA, 12.5 μL of 2× reaction buffer, 1 μL of reverse transcriptase, 1 μM of forward and reverse primer, and water to a total of 25 μL. The thermocycler protocol followed a previously described protocol with the reverse transcription (RT) step held at 56 °C for 20 min, followed by a denaturation step at 94 °C for 2 min, followed by 45 cycles of 94 °C for 15 s, 56 °C for 30 s, and 68 °C for 1 min, with a 5-min extension period at 68 °C [17].

PCR products were visualized on a 2% agarose gel stained with ethidium bromide. A positive control, an RdRp sequence from human coronavirus 229E in a p-GEM T-Easy plasmid (Promega, Madison, WI, USA), was run with each set of reactions. Positive samples with the appropriate amplicon size were purified and sequenced at RIBSP using the Genetic Analyzer 3130xl (Thermofisher, Waltham, MA, USA) with a Big Dye Terminator Cycle Sequencing kit, v. 3.1 (Thermofisher). Electropherograms were inspected in Geneious v11 [18]. A total of 12 RdRp sequences were newly generated from this study. To further understand the evolutionary relationships of these viruses, we analyzed novel bat coronavirus sequences in combination with 2811 RdRp sequences of coronavirus from different host species worldwide, representing the three genera: *Alpha-, Beta-*, and *Gamma-coronaviruses*. Global RdRp sequences were downloaded from National Center for Biotechnology Information sequence database (GenBank) and aligned using Transalign [19]. This large dataset was manually aligned and further down-sampled to 248 sequences to reduce redundant and similar sequences. Maximum-likelihood phylogeny of the partial RdRp gene (460 bp) was reconstructed by RAxML; GTR + GAMMA was selected for the model of nucleotide substitution as it allows rate heterogeneity among sites, as implemented in Geneious v11 [20]. Branch support was assessed using 1000 bootstrap replicates; bootstrap values greater than 50% were indicated at major nodes.

## 3. Results and Discussion

A total of 200 bat guano samples were collected from three sites: Kepterkhan tunnel (*n* = 101), Qaraungir cave (*n* = 50), and Ungirli cave (*n* = 49). Each cave was occupied by two bat species, the dominant species being *Myotis blythii* (lesser mouse-eared bat) and the more infrequent species being *Hypsugo savii* (Savi’s pipistrelle). Overall, 25 (12.5%) of all guano samples screened were positive for coronaviruses: Qaraungir cave with the highest percent positive (24%) and Kepterkham tunnel with the lowest (6.9%) (Table 1).

**Figure 2:**
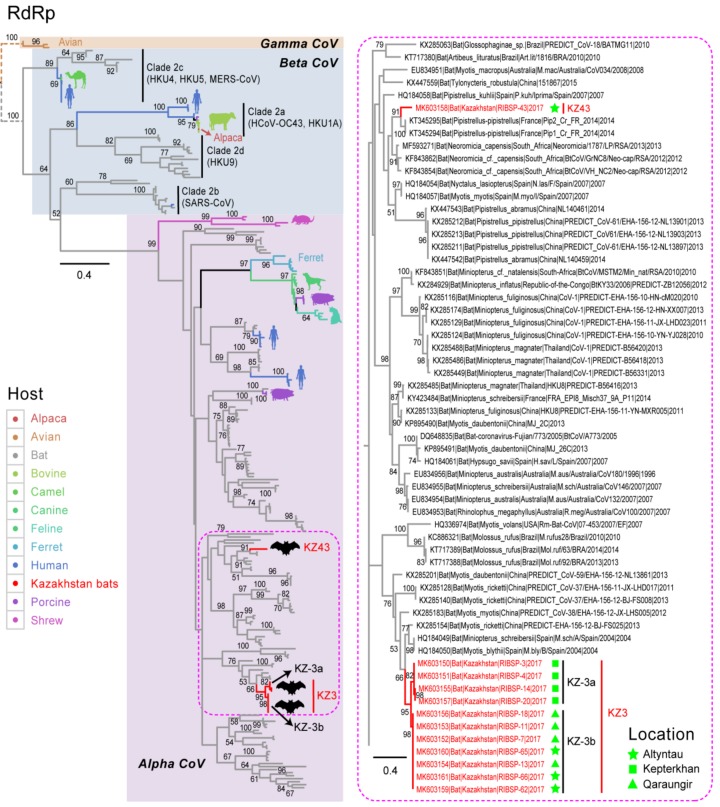
Phylogenetic relationships of the RNA-dependent reverse polymerase (RdRp) gene sequences of coronavirus, inferred using the maximum-likelihood method with the GTR + GAMMA model in RAxML. Representative virus isolates from *Alpha*-, *Beta*-, and *Gamma*-*coronavirus* (CoV) were included in the analysis. Colored branches and symbols denote viruses collected from different hosts. New CoV sequences generated from this study are marked by red branches. The dotted box indicates Kazakhstan coronavirus lineages (KZ3 and KZ43) described in this study and their closely related viruses isolated worldwide. The inset displays detailed strain names of the Kazakhstan coronaviruses and their closely related viruses. Cave locations are denoted by different shapes in green. Bootstrap support values greater than 50% are displayed at major nodes. The scale bar indicates the number of nucleotide substitutions per site.

Sequence data were successfully generated for 12 of the 25 PCR positive samples. Our RdRp phylogeny demonstrates that all 12 new CoV sequences (GenBank Accession MK603150–MK603161) from Kazakhstan bats belong to the genus *Alpha*-CoV (Figure 2; Figure S1, Supplementary Materials). The *Alpha*-CoV genus comprises a large number of coronaviruses from diverse hosts, including bats, shrews, dogs, cats, ferrets, pigs, and humans. In Kazakhstan bats, the new CoV sequences were found to be segregated into two different groups. The majority (11 sequences) of the Kazakhstan CoVs formed a strongly monophyletic single clade (bootstrap (BS) = 95%), referred to here as “KZ3”, with the nucleotide sequence identities ranging from 94.7% to 100%. The KZ3 clade was a sister group to three bat CoV sequences from Spain (*Miniopterus schreibersii* and *Myotis blythii*) and China (*Myotis ricketti*). Within the KZ3 clade, two smaller co-circulating lineages (KZ-3a and KZ-3b) were formed that are also strongly monophyletic (BS = 82% and 98%, respectively). The KZ-3b sub-lineage contained seven bat sequences (RIBSP-7, 1, 13, 18, 62, 65, and 66) with 100% nucleotide similarity; however, the samples were collected from two different sites. Bat KZ-3b sequences (RIBSP-7, 11, 13, and 18) were collected from the Qaraungir cave, whereas bat KZ-3b sequences (RIBSP-62, 65, and 66) were collected from the Altyntau cave. This suggests the Kazakhstan bats residing in two different caves appear to harbor highly similar CoV strains.

The KZ43 group contains only one Kazakhstan sequence (RIBSP-43) from the Altyntau cave, which is markedly divergent (nucleotide identities: 70.2–72.9%) from the KZ3 sequences. The RIBSP-43 sequence is most closely related with *Pipistrellus pipistrellus* CoV (nucleotide identity: 88.1%) described from France in 2014. These sequences in turn grouped with diverse bat species from broad geographical regions including Spain (*Nyctalus lasiopterus* and *Myotis myotis*), China (*Pipistrellus pipistrellus*), and South Africa (*Neoromicia* cf *capensis*). Taken together, our results indicate that Kazakhstan bats may harbor a wider diversity of *Alpha* CoV, possibly by means of the regional and intercontinental spread of the virus. Although the pooled feces prevented attribution to a singular species, the two lineages of *Alpha* CoV may be derived from the different species of bats, *Myotis blythii* and *Hypsugo savii*. KZ3 is similar to *Myotis* coronavirus sequences, while the KZ43 is most similar to a *Pipistrellus* sequence and *H. savii* is a pipistrelle bat. Our approach was opportunistic sampling in three sites, and the total sample size was 200, limiting the conclusions we can make about bat-borne coronaviruses in central Asia; however, this study provides a baseline for future studies.

**Figure 3:**
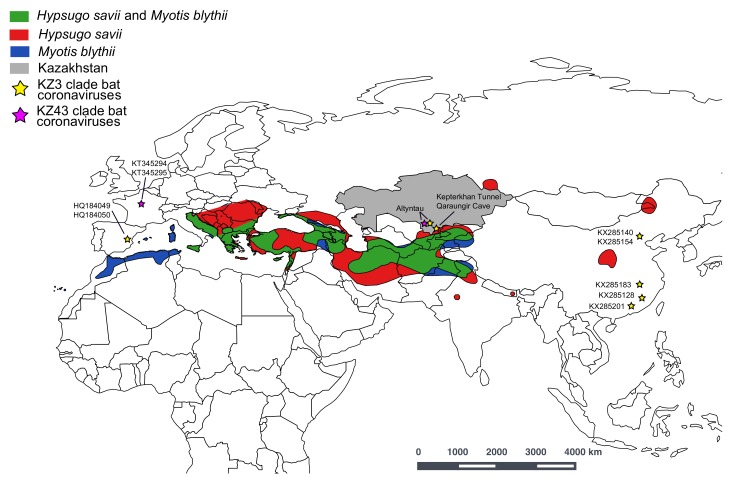
A map of the geographic distribution of *Myotis blytii* and *Hypsugo savii* species and where their ranges overlap (created in QGIS). The locations of the KZ3 bat coronavirus monophyletic clade (bootstrap (BS) = 76%) are presented on the map (yellow star), including Kazakhstan bat coronaviruses (KZ-3a and KZ-3b: MK603150–MK603157 and MK603159–MK603161), bat coronaviruses from China (KX285128, KX285140, KX285154, KX285183, KX285201), and bat coronaviruses from Spain (HQ184049, HQ184050). The locations of the KZ43 monophyletic clade (BS = 91%) are also listed on the map (purple star), including the Kazakhstan bat coronavirus (KZ43: MK603158) and bat coronaviruses from France (KT345294–KT345294).

Intense coronavirus surveillance is ongoing in China since the SARS-CoV outbreak, and it was demonstrated that co-roosting can maintain all strains with the necessary components to make SARS [10]. There is a paucity of bat-borne virus surveillance efforts across central Asia, even though this region is one of the largest grassland biomes in the world. This area is also of interest because it is where the Eurasian and Asian bat populations may be panmictic, as seen in other bat species with large distributions [21,22]. The range of *M. blythii* and *H. savii* is extensive, with both species found from China and northern India through to Spain, and these populations may be co-roosting at multiple sites due to the paucity of roosting areas in the steppe [23,24] (Figure 3). *Myotis blythii* is considered an occasional migrant, with movement of greater than 450 km recorded. The migratory behavior of *Hypsugo savii* is not known [25]. The potential panmixia and co-roosting of these bat species may lead to a similar mixing of viruses. These results provide a foundation to study bat-borne coronaviruses in Kazakhstan and highlight the need for collaborative efforts in understudied countries to increase integrated surveillance capabilities toward better monitoring and detection of infectious diseases.

## Figures and Tables

**Table 1:** The sampling collections of bat guano and PCR positives for coronavirus in Kazakhstan. GIS—geographic information system; N—north; E—east.

Sample Collection Site	GIS Coordinates	Samples Tested	Positives (%)
Kepterkhan tunnel, Tulkibas rayon, Turkestan oblast	N 42°32′ 36.5″ E 70°06′ 46.9″	101	7 (6.9%)
Qaraungir cave, Tulkibas rayon, Turkestan oblast	N 42°39′ 04.7″ E 69°54′ 02.6″	50	12 (24%)
Ungirli cave, Altyntau, Sozak rayon, Turkestan oblast	N 44°06′ 59.4″ E 67° 59′ 47.9″	49	6 (12.2%)

## Supplementary Materials

The following are available online at https://www.mdpi.com/1999-4915/11/4/356/s1: Figure S1. Phylogenetic relationships of the RdRp gene sequences of coronavirus, inferred using the maximum-likelihood method with the GTR + GAMMA model in RAxML. Representative virus isolates from *Alpha*-, *Beta*-, and *Gamma-coronavirus* were included in the analysis. Colored branches and symbols denote viruses collected from different hosts. New CoV sequences generated from this study are marked by red branches. Bootstrap support values greater than 50% are displayed at major nodes. The scale bar indicates the number of nucleotide substitutions per site.
