# Supplementary figures and images for: Discovery and Characterization of Novel Bat Coronavirus Lineages from Kazakhstan
Source: Viruses. 2019 Apr 17;11(4):356. doi: 10.3390/v11040356 (PMC6521082; doi:10.3390/v11040356)

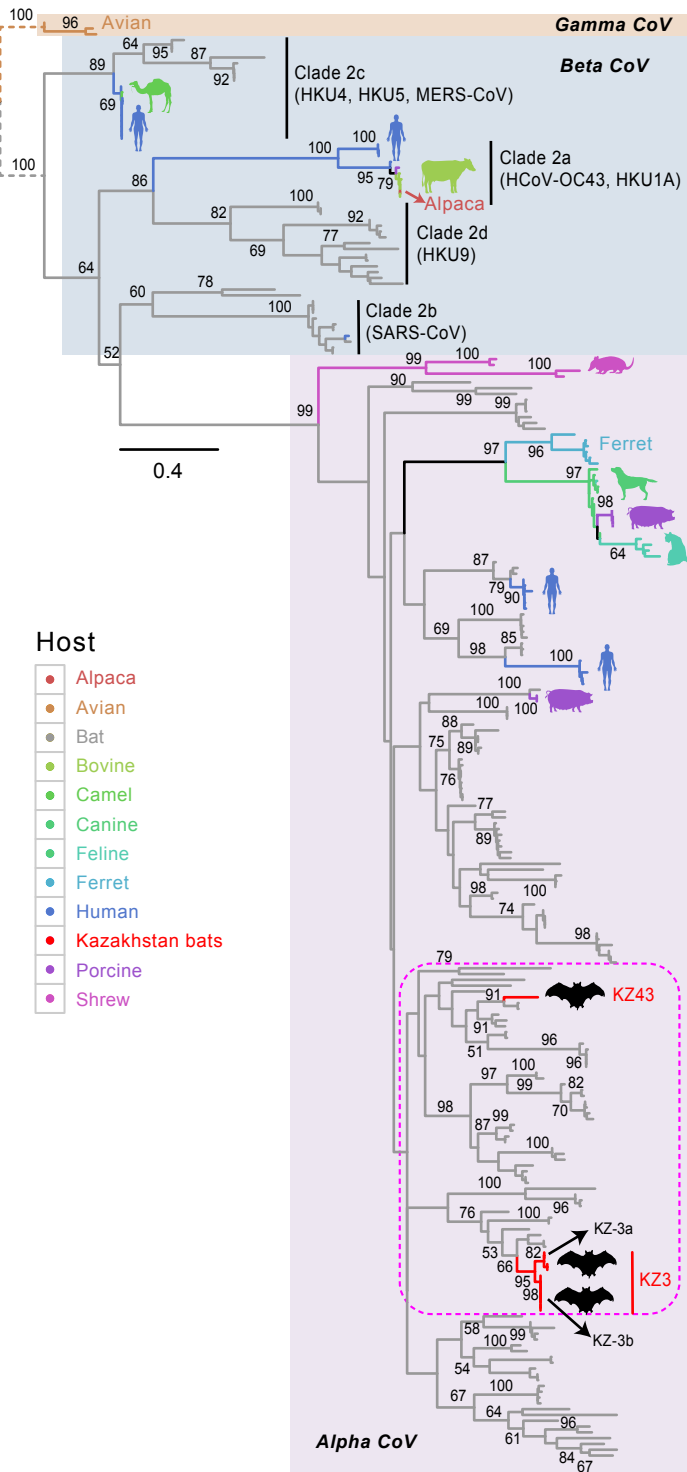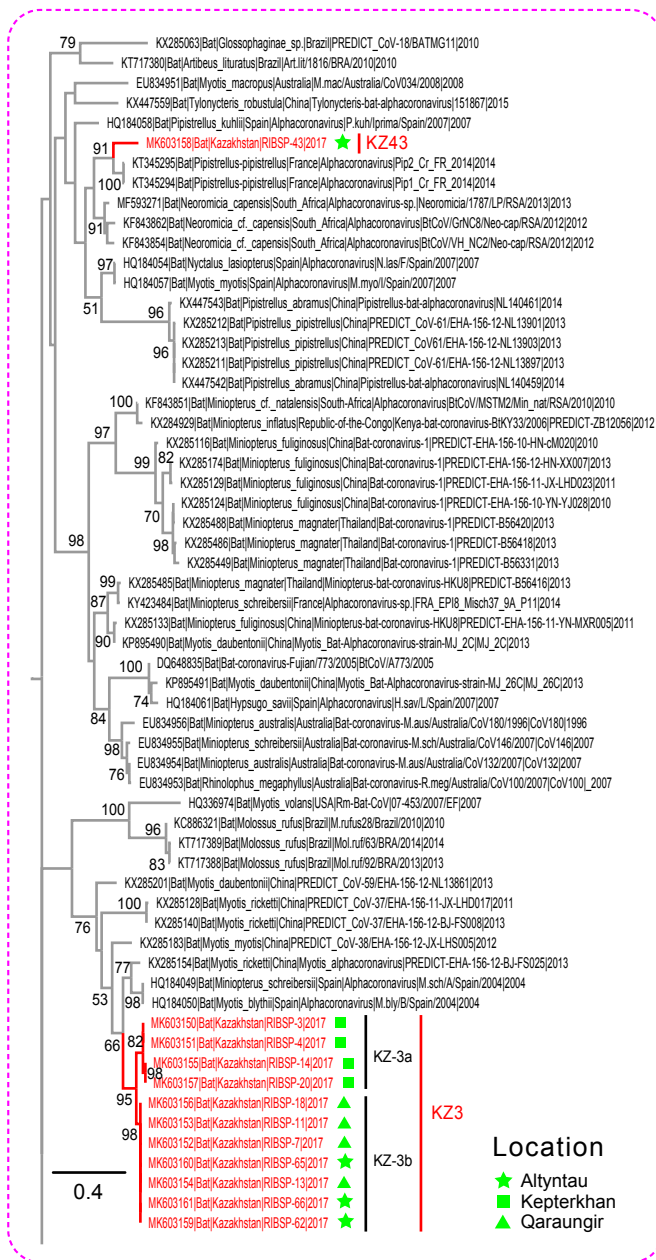

Supplement: Supplementary file 1 [file viruses-11-00356-s001.pdf]
